# Oncolytic viruses for cancer immunotherapy

**DOI:** 10.1186/s13045-020-00922-1

**Published:** 2020-06-29

**Authors:** Otto Hemminki, João Manuel dos Santos, Akseli Hemminki

**Affiliations:** 1 Division of Urologic Oncology, Department of Surgical Oncology, Princess Margaret Cancer Centre, University Health Network and University of Toronto, Toronto, Ontario Canada; 2 Cancer Gene Therapy Group, Translational Immunology Research Program, University of Helsinki, Helsinki, Finland; 3 Department of Urology, Helsinki University Hospital, Helsinki, Finland; 4 TILT Biotherapeutics Ltd, Helsinki, Finland; 5 Helsinki University Hospital Comprehensive Cancer Center, Helsinki, Finland

**Keywords:** Adenovirus, Oncolytic, Tumor, Cancer, Immunotherapy, Review, Immunology, Immune system, Immunosupression

## Abstract

In this review, we discuss the use of oncolytic viruses in cancer immunotherapy treatments in general, with a particular focus on adenoviruses. These serve as a model to elucidate how versatile viruses are, and how they can be used to complement other cancer therapies to gain optimal patient benefits. Historical reports from over a hundred years suggest treatment efficacy and safety with adenovirus and other oncolytic viruses. This is confirmed in more contemporary patient series and multiple clinical trials. Yet, while the first viruses have already been granted approval from several regulatory authorities, room for improvement remains.

As good safety and tolerability have been seen, the oncolytic virus field has now moved on to increase efficacy in a wide array of approaches. Adding different immunomodulatory transgenes to the viruses is one strategy gaining momentum. Immunostimulatory molecules can thus be produced at the tumor with reduced systemic side effects. On the other hand, preclinical work suggests additive or synergistic effects with conventional treatments such as radiotherapy and chemotherapy. In addition, the newly introduced checkpoint inhibitors and other immunomodulatory drugs could make perfect companions to oncolytic viruses. Especially tumors that seem not to be recognized by the immune system can be made immunogenic by oncolytic viruses. Logically, the combination with checkpoint inhibitors is being evaluated in ongoing trials. Another promising avenue is modulating the tumor microenvironment with oncolytic viruses to allow T cell therapies to work in solid tumors.

Oncolytic viruses could be the next remarkable wave in cancer immunotherapy.

## Background

Cancer is becoming a leading cause of death globally. Eighteen million new cancers are diagnosed every year causing almost 10 million deaths (International Agency for Research on Cancer (IARC), 2019). There has been significant progress in the prevention and diagnosis of cancer, but the incidence and mortality are still increasing [1–3]. Conventional therapies, such as surgery, chemotherapy, hormonal therapies, targeted therapies or radiation therapy, deliver limited durable responses in a great majority of patients with advanced cancers [4, 5]. Hematological and testicular malignancies are some of the few exceptions where current therapies can be curative even in metastatic cases [6–8].

Cancer is a genetic disease and it represents a range of manifestations. The principles of tumorigenesis are however similar across different tumors and relatively well characterized. In brief, frequent mutations occur during cell divisions or due to exogenous factors such as radiation or other carcinogens. Most of these mutations are corrected by specialized intracellular proteins. If such mechanisms are unsuccessful, mutated cells are generally cleared by apoptosis. The vast majority of mutations do not help the cell to gain cancerous properties (passenger mutations). In contrast, driver mutations provide exclusive abilities to tumor cells, such as cell death resistance or metastatic capacity, for example [9, 10]. Most of these mutated cells are, however, recognized by our immune system and destroyed before clinical detection. Accumulating evidence supports the notion that a dysfunctional immune system is intimately associated with tumor development, progression, and recurrence [11]. Also known as immunosuppression, this phenomenon is actively propagated by cancer cells either directly or through the tumor microenvironment [12]. This understanding has galvanized the interest in the development of immunotherapies, which aims at modifying and activating immune cells to attack cancer cells. The approach is rational as our immune system has been trained to detect, destroy, and memorize non-self patterns. By definition, all cancer cells have multiple mutations causing non-self structures that can potentially be detected by our immune system [9].

The concept of immunotherapy has been acknowledged already for centuries (Table 1). The relationship between microbial infections and spontaneous tumor regressions has been reported several times in the literature [13]. Probably the first evidence was the Ebers papyrus (1550 bc), one of the oldest and most important medical documents of ancient Egypt. The physicians of Egyptian pharaoh Imhotep (2600 bc) used poultice, followed by incision, for the treatment of tumors. This facilitated the development of infections which helped to cause regression of tumors [14]. In 1320, Peregrine Laziozi was affected by cancer of the tibia requiring amputation. Unfortunately, local recurrence and progression were later observed and the tumor finally grew through his skin causing infection. Later, to everyone’s astonishment, the tumor disappeared and no relapse was observed. This phenomenon is today known as the St. Peregrine tumor [15].

**Table 1:** A timeline including some key steps in development of cancer treatments

Other cancer treatments*		Cancer Immunotherapy
Surgery	2600 BCE	Use of poultice (pharaoh Imhotep’s physicians)
Surgery under ether anesthesia	1840s CE	Purposeful infection of tumors
Radiotherapy	1890s	Coley’s toxins (deactivated bacteria) were injected to tumor
Hormonal therapy (estrogen, castration), chemotherapy (nitrogen mustard, antifolates)	1900–1940s	Case reports of tumor regression after natural viral infections
Linear accelerator for radiotherapy, combination chemotherapy	1950s–1970s	Hundreds of case series treating cancer with multiple viruses (e.g., varicella, measles, vaccinia, West Nile, adenovirus, mumps) BCG adopted in bladder cancer
Stereotactic radiotherapy, antiestrogens	1980s	Adoptive T cell transfer, cytokine therapies (e.g., IFN-alpha and IL-2)
Mini-invasive surgery, monoclonal antibodies (rituximab, trastuzumab)	1990s	HD-IL-2 approved by the FDA
Antiangiogenic therapies (bevacizumab), kinase inhibitors (imatinib)	2000s	First oncolytic adenovirus (H101) approved in China
Small molecular inhibitors of various proteins	2010–	Cellular immunotherapy (sipuleucel-T, TCR, CART), six different checkpoint inhibitors, oncolytic virus (T-vec)

*Many treatments in this column have also immunological properties (e.g., rituximab, trastuzumab, chemotherapy, and radiation therapy)

In the seventeenth and eighteenth centuries, various forms of immunotherapy became widely used. In the eighteenth and nineteenth centuries, septic dressings enclosing ulcerative tumors were sometimes used for cancer treatments. Surgical wounds could be deliberately left open to enable the development of infection as purulent infections were suggested helpful [15]. One of the more detailed case series, indicating several responses, was reported by surgeon William B. Coley. He treated cancer patients with a bacterial lysate (heat-killed Streptococcus pyogenes and Serratia marcescens), also known as the Coley’s toxin [16].

Reports of viruses having therapeutic benefits in cancer started appearing early last century with multiple reports of leukemia patients becoming disease-free after viral infections [17]. Typically, the reported patients were young and the remissions were short-lived lasting for 1 or 2 months [18]. These observations did not go unnoticed by the medical community, who subsequently begun utilizing viruses for the treatment of cancer. Especially during the 1950s and 1960s, multiple wild type viruses (e.g., hepatitis, Epstein-Barr, West Nile, Uganda, dengue, yellow fewer) were used to treat different cancers in hundreds of case series. Results were variable and occasionally poorly documented [17]. However, during this time, it was becoming clear that most wild type viruses lacked efficacy or safety. Some of the more promising results with tolerable side effects were associated with adenoidal-pharyngeal-conjunctival virus [19–22], nowadays known as the adenovirus. For example, in 1956, 30 women with advanced epidermoid carcinoma of the cervix were treated with adenovirus. Intra-arterial, intravenous, and intratumoral administration was used. Within 10 days, two-thirds of the patients showed necrosis in their tumors and most remarkably, it appeared to be restricted to the cancerous tissue. No safety problems were reported in the use of this wild type virus, suggesting a degree of natural tumor tropism.

Generally, however, virotherapy received little attention and in the 1970s and 1980s, the regulatory aspects of clinical trials with living pathogens became stricter. In turn, chemotherapies, radiation therapy, hormonal therapy, targeted, and antiangiogenic therapies all became mainstream. It took more than three decades before viruses re-emerged, this time as “oncolytic viruses” [23, 24]. An oncolytic virus is a virus that infects and lyses (breaks down) cancer cells but not normal cells. Oncolytic viruses can occur naturally or can be made in the laboratory by modifying natural viruses. These modifications started a new era of less toxic cancer targeted virus-based therapies [25].

Rapid increases in molecular biotechnology techniques provided means to develop novel strategies to harness the immune system for cancer therapy. Currently, a number of approaches, including adoptive cell therapies, monoclonal antibodies, checkpoint inhibitors, and oncolytic viruses constitute the most prominent advancements in cancer treatment due to the capacity to provide durable and effective clinical responses in cancer patients [13]. Conventional treatments such as radiation and chemotherapy treatments also seem to have immunomodulatory effects not recognized before [26, 27].

However, it is essential to note that presently therapeutic benefits are restricted to a limited fraction of patients treated with immunotherapy. In particular, solid cancers generally contain a suppressive tumor microenvironment that inhibits T cell activity and supports tumor progression [28]. In addition, new immunotherapy treatments have led to the occurrence of new immunological adverse events, including cytokine storm and autoimmune events. Considering these challenges, further alterations to these therapeutic strategies are needed. In addition to new immunological treatment strategies, we also need better understanding of individual immune environments to provide maximal patient benefit.

Our aim in this review is to present the current possibilities that oncolytic viruses have to offer. We concentrate on adenoviruses that are the most widely studied virus type. We start by describing general adenovirus biology and describe then some typical modifications that can be used to generate better anti-cancer capabilities. Finally, we describe clinical studies with oncolytic adenoviruses and describe three different types of oncolytic viruses that are already regulatory approved to treat cancer. The aim is to provide a general overview on the field and we acknowledge that many viral constructs, especially with preclinical studies only, are not covered. However, recruiting or completed oncolytic adenovirus trials (source clinicaltrials.org) give a good general view of where the field is today. Finally, we conclude by addressing some future perspectives.

## Main text

### Structure, function, and immunogenic cell death: adenovirus as an example

Adenovirus biology has been investigated in detail and it is well understood. Adenoviruses can be used as an example virus when describing oncolytic viruses as a whole. While there are naturally many differences with different viruses, many factors are similar to some degree, however not all viruses are naturally oncolytic.

To date, 57 distinct adenovirus serotypes are described and classified into 7 subgroups: A to G [29, 30]. While adenoviruses trigger common-flu type infections, these represent one of the most versatile platforms for cancer therapy. In particular, serotype 5 (group C) is the most commonly used backbone for oncolytic virus design [29]. Its structure encompasses an icosahedral shaped capsid (composed mainly of hexon, penton, and fiber proteins) surrounding a non-enveloped double-stranded DNA [31]. Adenoviruses have the ability to infect cells independently of their division status.

Infection of tumor cells initiates with the attachment of the virus fiber knob to receptors located on the surface of tumor cells. This interaction is mediated by different receptors depending on the serotype of the virus. For example, serotype 5 adenoviruses bind preferentially to the coxsackie- and adenovirus receptor CXADR [32], while serotype 3 adenoviruses bind desmoglein-2, CD46 or CD80/86 [33–35]. Some of these receptors are frequently found on cancer cells, while unfortunately some, such as CXADR, are downregulated in many advanced tumors [30]. A second interaction between the virus’ penton proteins and tumor cell integrins occurs, resulting in virus internalization [36]. Adenoviruses can use also several other receptors as shown in Table 2. A multistep process takes the virus DNA into the nucleus, where transcription of early viral proteins (E1–E4) starts. Following expression of late proteins, thousands of new viral progeny emerges, disrupting the cell membrane after a few days and the newly formed viruses infect new cells, until the immune system eventually stops this process [50].

**Table 2:** Entry receptors for adenoviruses [37]

Receptor	Ligand	Reference
Primary receptors		
CXADR (coxsackie-adenovirus receptor)	Ad5 knob (and other groups A, C, D, E, and F)	[38]
CD46	Ad3 knob (and other groups B and D)	[39]
Desmoglein-2	Ad3 knob (and other group B)	[40, 41]
CD80/CD86	Ad3 knob (and other group B)	[33, 37]
Other receptors		
Cellular integrins	Ad capsid (penton base) or Arg-Gly-Asp (RGD) modifications	[42]
Sialic acid	Ad 37, 8, 19a (group D)	[43]
MHC-I (major histocompatibility complex class I)	Ad5	[44]
Vascular adhesion molecule 1 (VCAM-1)	Ad5	[45]
Heparan sulfate glycosaminoglycans (HS-GAGs)	Ad5	[46]
Dipalmitoyl phosphatidylcholine (DPPC)	Ad5 hexon	[47]
Blood coagulation factor F(X)	Ad 5 hexon	[48, 49]

**Fig. 1:**
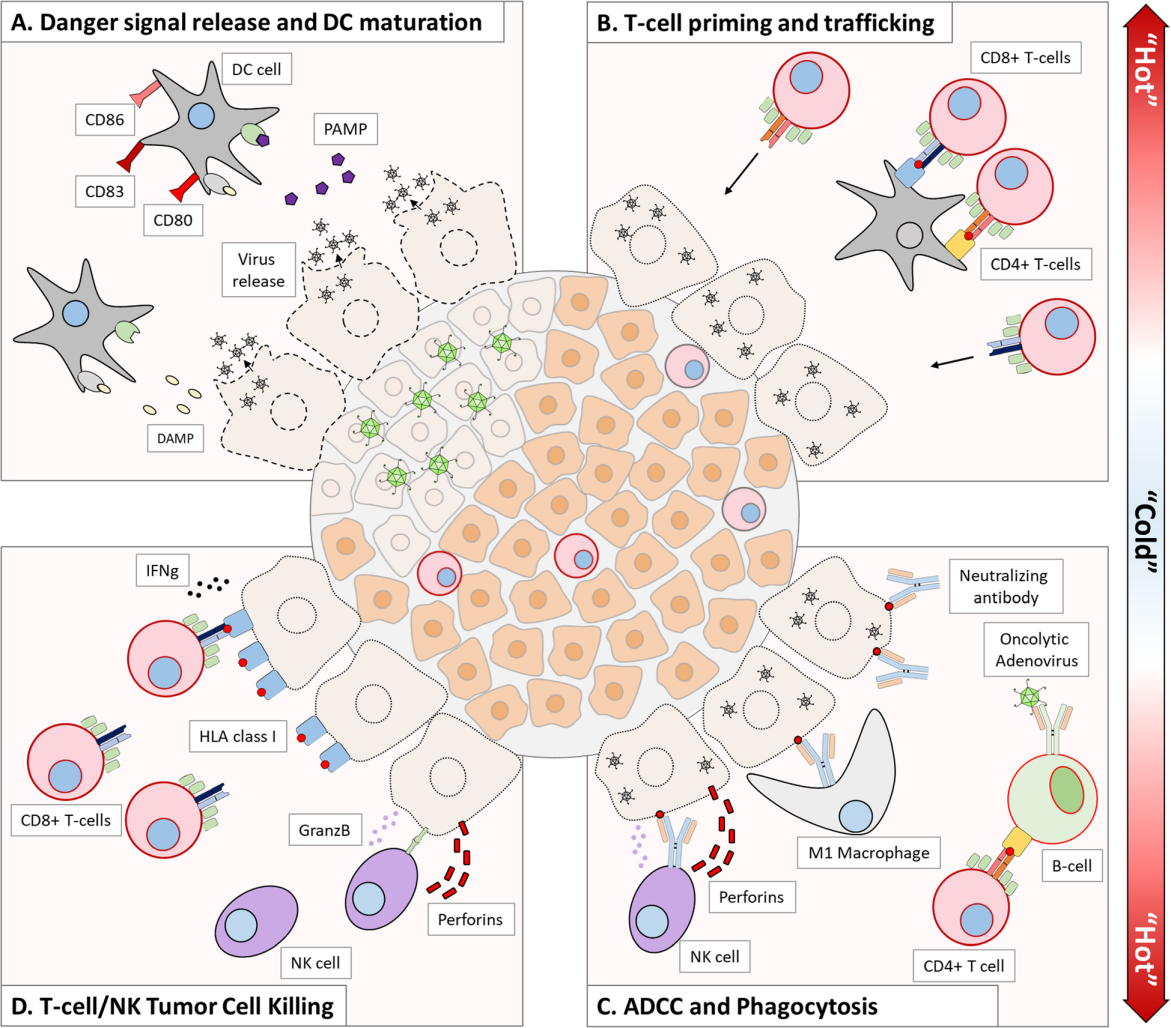
Activating the immune system for cancer rejection with oncolytic virus therapy. The tumor microenvironment of advanced cancers is “cold” due to the lack of immunological activity. Oncoytic virus therapy restores the immunological activity of immune tumor infiltrates. **a** Danger signal release and DC maturation. Oncolytic adenoviruses infect tumor cells and cause oncolysis, releasing new virus progeny but also DAMPS and PAMPS, which will activate nearby dendritic cells and foster their maturation by upregulating co-stimulatory markers, such as CD80, CD83, and CD86. **b** Mature dendritic cells will process tumor debris and present tumor-associated and virus antigens to local and distant T cells. Concurrently, the ongoing virus infection attracts T cells to the tumor site. **c** The activation of B cells by CD4+ T cells or BCR-virus interaction causes the release of neutralizing antibodies, which mark infected tumor cells for ADCC by NK cells, or phagocytosis by M1 macrophages. **d** CD8+ T cells and NK cells destroy infected and non-infected tumor cells through INFg/GranzB and GranzB/Perforins, respectively. The oncolytic adenovirus infection also upregulates class I HLA in tumor cells, allowing for increased exposure to CD8+ T cells. Overall, the components of this modulation allow the tumor microenvironment to become “hot” with increased immunological activity. DAMP danger-associated molecular patterns, PAMP pathogen-associated molecular patterns, HLA human leukocyte antigen, BCR B cell receptor

Although not discovered until the treatment of patients, such cell lysis is a highly immunogenic process [51]. This factor is of pivotal importance considering that most cancers seem to be able to hide from our immune system. Immunogenic cell death reveals multiple tumor-associated antigens for presentation to the immune system via activated mature dendritic cells. High numbers of virus genomes activate immunological danger signaling through damage-associated molecular pattern (DAMP) and pathogen-associated molecular pattern (PAMP) receptors. These processes form a recipe that retargets the adaptive immune system, including cytotoxic CD8+ T cells and helper CD4+ T cells, towards the tumor, thus lifting local immunosuppression [52]. Of note, anti-adenoviral T cell immunity is polyfunctional rendering increased quality to the overall ongoing antitumor response [53]. Simultaneously, adenovirus infection also mediates activation of natural killer cells further contributing for the antitumor immune response [54]. The generation of antiviral antibodies equally stimulates the response by triggering antibody-dependent cytotoxicity [55] (Fig. 1).

### Modern oncolytic viruses

Today, adenoviruses, herpes viruses, measles viruses, coxsackie viruses, polioviruses, reoviruses, poxviruses, and Newcastle disease viruses, among others, are some of the oncolytic viruses under preclinical and clinical development for cancer therapy [56]. Tumor-preferential replication can be “natural” given the defective viral sensing mechanisms of most cancer cells [57]. Some of the cancer cells also harbor increased expression of viral entry receptors, and some viruses do not appear to need specific receptors for entry [58]. Abnormal function of intracellular signaling pathways such as interferon can be exploited by some viruses [58]. Like many other viruses, adenoviruses are naturally prone to replicate aggressively in tumor cells and, their wild type versions could in theory be used in cancer treatments, as was done in historical series [17, 19–22]. However, better patient outcomes are expected by rational design of viruses rendering them tumor selective (“oncolytic”). In recent years, adenoviruses have been extensively altered to merge high antitumor potency with minimal toxicity [25].

Existing molecular biology techniques allow us to (a) select entry receptors highly expressed on tumors, (b) refine safety by restricting replication to cancer cells, and (c) insert specific therapeutic transgenes for increased efficacy. These approaches are discussed below and summarized in Table 3.

**Table 3:** Examples of viral modifications in oncolytic adenoviruses

Modification	Reference
Enhancing tumor tropism	
RGD modification	[59–61]
5/3 chimerism	[62, 63]
Fully serotype 3, Ad3/Ad11p	[30, 64, 65]
Safety, restricting replication to cancer cells	
E1A gene 24-base pair deletion	[61, 66, 67]
hTERT promoter	[64, 68]
p53 promoter	[69]
CEA promoter	[70]
PSA promoter	[71]
E2F promotor	[72]
Cox2l promoter	[73]
Transgenes, enhancing efficacy	
Cytosine deaminase and thymidine kinase	[74]
Ganciclovir and/or 5-fluorocytosine (5-FC) prodrugs	[75]
GMCSF	[66, 67, 72, 76, 77]
CD40L	[78, 79]
hNIS	[80]
TNFalpha and interleukin 2	NCT04217473
CD40L and 41BBL	NCT03225989
PH20 hyaluronidase	NCT03284268
Anti-CTLA4	[81]
IL-12 and decorin	[82]
OX40L	[83]
EGFR	[84]
FR-a	[85]
FAP	[10]
CD44v6	[86]

#### Enhancing tumor tropism

Effective entry of oncolytic viruses into tumor cells is a prerequisite for subsequent oncolysis. Conversely, low receptor expression can be a limiting factor. To avoid the conundrum of low expression of CXADR on tumors cells, serotype 5 adenoviruses can be modified to contain arginylglycylaspartic acid (RGD) peptides in their fiber knobs. Such modification has been shown useful to increase efficacy and reduce the toxicity of adenoviruses [59, 60]. Clinical implementation of such modified oncolytic adenoviruses, such as DNX-2401 (an serotype 5 adenovirus with an RGD modification), has shown promising results in a phase I clinical study where 20% of glioma patients showed durable responses [61].

Likewise, replacement of the serotype 5 fiber knob with one belonging to the serotype 3 has provided substantial improvements in antitumor efficacy, while retaining the appealing systemic kinetics and safety of the ubiquitous serotype 5 [62, 63]. Capsid modification (e.g., 5/3 chimerism) allows partial overcoming of pre-existing neutralizing antibodies against Ad5 [72]. Of note, the antibody question is complex, and in fact baseline neutralizing antibody titers have not prevented antitumor efficacy in humans [76, 87]. The large amounts of virus that are produced by tumors into the bloodstream might simply overcome neutralizing antibodies producible into blood. In wild type adenovirus infections, only small amounts of adenovirus enter the blood. Neutralizing antibodies are designed to block such virus, not the huge numbers produced by tumors infected with oncolytic virus. Nevertheless, in epidemiological analysis, lack of antibodies at baseline impacted survival statistically significantly [76], but not in a clinically meaningful manner, as responses and long survival could be seen regardless of baseline antibody titers. Interestingly, sequential intravenous treatments by changing the virus or serotype might make a difference [88].

As expression of the Ad5 receptor CXADR appears limiting for efficacy in the context of advanced tumors, fully serotype 3-based oncolytic adenoviruses have been constructed [64]. This virus enters through non-CXADR-mediated mechanisms including desmoglein 2, which is highly expressed in advanced solid tumors [30, 35, 64, 78]. Good safety and signs of efficacy were seen in patients treated with a fully serotype 3 adenovirus [64]. Furthermore, a direct evolution method [65] was used to generate an Ad3/Ad11p chimeric virus which has been used in multiple trials under the name ColoAd1 [89].

#### Safety, restricting replication to cancer cells

To achieve efficient dissemination of input virus and to minimize virus-related adverse events, adenoviruses have been modified to achieve increased tumor selectivity. The strategies employed include transcriptional control of adenovirus early proteins such as the E1A or E1B. An E1A gene 24-base pair deletion produces a mutated E1A protein which cannot bind to the retinoblastoma protein, thus preventing healthy cells from entering in synthesis (“S”) phase. This blocks adenovirus DNA replication in quiescent normal tissues [61, 66]. In contrast, the replicative potential remains intact in tumor cells because ubiquitous defects in the p16/Rb pathway ensure that cancer cells permanently stay in synthesis phase [90].

The fact that tumor cells contain several active oncogenes led to the realization that their resulting proteins could be harnessed to control transcription of adenovirus DNA. For example, telomerase activity is a known feature of cancer cells, while activity in healthy cells is minimal [30, 91]. Therefore, adenovirus replication has been successfully placed under the control of a human telomerase reverse transcriptase (hTERT) promoter showing antitumor efficacy in advanced cancers [64, 68]. Similarly, p53 [69], carcinoembryonic antigen (CEA) [70] and prostate-specific antigen (PSA) [71] have been utilized to control expression of early adenovirus proteins. Due to Rb/p16 pathway defects, cancer cells feature high levels of intracellular free E2F, which can be used for tumor specificity when the E2F promotor is inserted to control viral replication [72].

#### Transgenes, enhancing efficacy

The nature of viruses allows them to hijack the host cell to produce virus proteins. This allows therapeutic exploitation with the insertion of therapeutic transgenes into the adenovirus genome. Before the recognition of oncolytic adenoviruses as immunotherapy, one of the most common modifications was the insertion of the cytosine deaminase and herpes simplex-derived thymidine kinase [74]. The combined administration of oncolytic adenoviruses with ganciclovir and/or 5-fluorocytosine (5-FC) prodrugs caused tumor cell death due to their conversion into cytotoxic compounds by transduced tumor cells [75].

More recently, the increased recognition of the immune system as an important component in the efficacy of oncolytic viruses led researchers to perceive oncolytic adenoviruses as potent vehicles for immune factors. Adding a granulocyte macrophage colony stimulating factor (GMCSF) cytokine transgene into the adenoviral genome is a commonly used modification. In this approach, virus replication is accompanied by GMCSF production, which results in the recruitment and maturation of dendritic cells (DCs), and subsequent priming of T cells with tumor-associated antigens released by oncolysis [92]. CGTG-102 (previously Ad5/3-D24-GMCSF, currently ONCOS-102), is an oncolytic adenovirus expressing GMCSF. Patient data confirms this notion, with reported increases in peripheral levels of T cells against tumor-associated antigens [66]. These finding suggest dendritic cell priming in humans as predicted by the established mechanism of action of GMCSF [93]. The increased CD8+ T cell infiltration found in tumor biopsies after treatment of advanced cancer patients with ONCOS-102 underlines the immunological potency of this approach [94]. However, the pleiotropic effects of GMCSF may endanger antitumor immunity as the cytokine can unwantedly stimulate myeloid-derived suppressor cells (MDSCs) and tumor-associated macrophages (TAMs), both known to inhibit T cell and natural killer cell (NK) activity [95]. However, emerging human data suggests that GMCSF producing viruses might be safe and effective [76, 77].

Beyond GMCSF, combined expression of IL-12 and decorin in an oncolytic adenovirus allowed the recovery of antitumor immunity in a poorly immunogenic murine breast cancer model, via cytotoxic T cell infiltration and transforming growth factor beta (TGFb) reduction [82]. Coexpression of CD40L and 4-1BBL by an oncolytic adenovirus has also shown promising results, due to its ability to promote the destruction of pancreatic tumors, through repolarization of the tumor microenvironment. Such polarization enabled release of T cell attractants and immune stimulatory cytokines, allowing potent antitumor T cell responses [96]. Production of OX40L mediated by an oncolytic adenovirus (d24-RGDOX) promoted increased tumor control via highly functional effector T cells and epitope spreading [83].

Also, antibodies can be inserted as transgenes to enhance the efficacy of oncolytic virotherapy. For example, anti-CTLA4, a checkpoint inhibitor, has been successfully inserted in an oncolytic adenovirus platform. Its usage in murine models and ex vivo cultures of cancer patient peripheral blood mononuclear cells (PBMCs) resulted in increased antitumor activity of T cells [81]. More recently, dual targeted antibodies targeting T cells and cancer-specific cell surface antigens such as epidermal growth factor receptor (EGFR) [84], FR-a [85], familial adenomatous polyposis (FAP) [10] and CD44v6 [86], have demonstrated promising preclinical results [84].

Moreover, also other approaches have been studied. These include arming with fusogenic molecules, antibodies, T cell engagers, and ion channels capable of concentrating radioiodine. While in these cases, the transgenes are not necessarily immunologically active, and the oncolytic platform results in immunostimulation. It is important to note that clinical data suggests that oncolytic adenovirus single-agent efficacy has often been somewhat limited. Several barriers that affect oncolytic adenovirus therapies have been suggested. These include antiviral interferons, which can be produced by the tumor stroma even if the cancer cells themselves lack such ability [50]. Other reasons include stromal barriers, hypoxia, hyperbaric, necrotic, and acidic areas [97, 98]. However, some of these hurdles have been addressed in redesigned adenoviruses conditionally replicating in response to hypoxic factors or acidic tumor microenvironments [99].

Alternatively, oncolytic adenoviruses have been armed with hyaluronidase [100], an enzyme that degrades hyaluronic acid which hampers virus dissemination. Notably, treatment of a number of preclinical in vivo tumor models allowed increased antitumor efficacy. Neutralizing antibodies remain a concern for oncolytic immunotherapies. However, the use of bispecific adapters to retarget antiviral neutralizing antibodies can offer an attractive approach to increase the effectiveness of oncolytic adenovirus therapy [101]. The coating of oncolytic adenoviruses with tumor derivatives has been reported to allow for successful delivery of particles into the tumor with potent antitumor responses [102].

### Advanced Therapy Access Program (ATAP)

Between 2007 and 2012, 290 advanced cancer patients were treated with 10 different oncolytic viruses (Table 4) totaling 821 treatments. A long-term follow-up of these patients has been published [5]. Treatments were given in the context of an individualized treatment program under the EU Advanced Therapies directive [5]. While many objective responses were seen, no definite conclusions regarding overall survival benefit can be drawn as no reliable control group was available. However, some case-control analyses that were performed suggest survival benefit [88].

**Table 4:** Viruses used in ATAP

	Serotype	Main target receptor	Tumor specificity	Arming
Ad5-D24-GMCSF [92]	5	Coxsackie virus and adenovirus receptor	24 bp deletion in E1A^1)^	GMCSF
Ad5-RGD-D24 [103]	5	Alpha-v-beta integrins	24 bp deletion in E1A^1)^	No
Ad5-RGD-D24-GMCSF [103]	5	Alpha-v-beta integrins	24 bp deletion in E1A^1)^	GMCSF
ICOVIR-7 [104]	5	Alpha-v-beta integrins	E2F1 promoter and 24 bp deletion in E1A^1)^	No
Ad5/3-Cox2L-D24 [73]	5	Desmoglein-2	Cox2L promoter and 24 bp deletion in E1A^1)^	No
Ad5/3-D24-GMCSF [88]*	5	Desmoglein-2	24 bp deletion in E1A^1)^	GMCSF
Ad5/3-hTERT-hCD40L [73]	5	Desmoglein-2	hTERT promoter^2)^	CD40L
Ad5/3-E2F1-D24-GMCSF [105]	5	Desmoglein-2	E2F1 promoter and 24 bp deletion in E1A ^1)^	GMCSF
Ad5/3-D24-hNIS [80]	5	Desmoglein-2	24 bp deletion in E1A^1)^	hNIS
Ad3-hTERT-E1A [64]	3	Desmoglein-2	hTERT promoter^2)^	No

^*^Ad5/3-D24-GMCSF, also known as CGTG-102, and later renamed ONCOS-102, has been subsequently used in several phase 1 and phase 2 clinical trials (www.targovax.com)

^1)^Replication in cells with a deficient Rb/p16 pathway (a hallmark of cancer)

^2)^Replication in cells with active telomerase (a hallmark of cancer)

While taking into account the limitations of nonrandomized data, some interesting findings emerged [5, 51, 64, 66, 80, 88, 92, 103, 104, 106]. One of the most important observations was that all of the administered viruses appeared quite safe in patients with advanced cancer. Good tolerability was seen across different serotype viruses, including various capsid modifications, and different immunological arming devices (i.e., GMCSF or CD40L). Concomitant low-dose cyclophosphamide and temozolomide were also well tolerated [107]. The former was used to reduce regulatory T cells. The latter aimed at increased induction of autophagy in infected cancer cells, as this appears to be an important mediator of oncolytic cell death. Moreover, virus replication could be increased by concomitant calcium channel blockage [108]. No treatment-related patient deaths were observed [63]. Typical flu-like symptoms, such as fever and fatigue, were observed in most patients a few days after treatment. These findings were confirmed in multiple subsequent clinical trials [61, 94, 109]. Flu-like symptoms and fever could be effectively reduced with acetaminophen (paracetamol).

### Systemic delivery of oncolytic virus

Biodistribution studies done with adenoviruses in rodents are unreliable as most animals lack entry receptors or their organ distribution is different from humans. From ATAP patients, we have been able to collect important information about virus biodistribution in humans [63]. Many humans have neutralizing antibodies against different adenovirus serotypes, although often at low baseline titer [64]. However, as hundreds of billions of viruses are given in a typical treatment, pre-existing antibodies may be unable to completely block intravenous delivery. In subsequent intravenous treatments with the same serotype, the situation is more unclear, which is one of the reasons intratumoral delivery is commonly used with oncolytic viruses. Of note, it has been proposed that antiviral immunity helps generate antitumor immunity [66].

It has been established in humans that adenovirus is able to travel through blood to metastases despite neutralizing antibodies [63]. For some viruses, the mechanism appears to relate to binding to blood cells [110]. Interestingly, adenoviruses in blood (qPCR data) were most often found from blood clots, while some patients had significant amount of virus in the serum compartment [64]. Also cancer patient’s antibody response varied [64]. Treatment responses or long survival are seen regardless of neutralizing antibody titers, although it should be noted that most patients were treated intratumorally [76].

Interestingly, we treated seven patients with the serotype 3 adenovirus using only intravenous administration. Signs suggesting virus replication were seen, including prolonged and/or rising virus titers in the blood. This was seen also with patients who had pre-existing antibodies against the virus. Also, 5 of the 6 evaluable patients showed signs of possible benefit. This data indicates that viruses might be able to enter tumors also via the intravenous route [64]. This was later confirmed in an autopsy study where non-injected tumors were shown to have oncolytic adenovirus [63].

### Viruses that have already received regulatory approval for the treatment of cancer

From a clinical standpoint, the use of viruses for cancer treatment in the modern era is in its infancy. Initially, wild type viruses were used, but this approach could result in adverse events caused by virus replication in normal tissues. Nevertheless, Rigvir (an ECHO-7 virus) [111], an oncolytic picornavirus with some innate tumor selectivity, was the first approved oncolytic virotherapy product for cancer approved 2004 in Latvia and later in a few other countries. The second oncolytic virus was rationally designed for tumor selectivity. Named H101 (Oncorine), this adenovirus has been used in China since 2005 for the treatments of solid tumors [112]. Of note, both of these viruses lack arming devices.

The acknowledgement that repurposing the immune system to exert antitumor functions could provide a promising approach to treat cancer enabled scientists to employ the immunological capabilities of oncolytic viruses [113]. For example, the addition of immunological transgenes such as the GMCSF has been a popular approach. Talimogene laherparepvec (also known as T-vec, Imlygic®) is a herpes simplex-1 virus encoding for GMCSF, and was one of the first oncolytic viruses designed to provide an immunological boost. Its clinical application eventually led to a randomized phase III clinical trial (OPTiM). In this trial stage IIIB/C and IV metastatic, unresectable melanoma patients receiving intratumoral T-vec had a 19.3% durable response rate of which more than 80% were complete responses [114]. The fact that subcutaneous administration of GM-CSF offered inferior efficacy (1.4% durable response rate, 0.7% complete response) led to approval by the Food and Drug Administration (FDA) in 2015, followed by European Medicines Agency (EMA) [114].

This landmark approval in Western countries encouraged optimism in the medical community to continue developing and improving oncolytic viruses for cancer therapy, including adenoviruses. Later, in a similar patient population, pembrolizumab was combined with the virus leading to responses in 62% of the patients, of which 33% were complete. As expected, a high presence of cytotoxic T cell infiltration was observed in the tumors following treatment [115]. A recent phase II clinical trial in advanced melanoma patients demonstrated that T-vec increased the response rate of ipilimumab as compared to ipilimumab alone (38% vs 18%, respectively) [116]. Of note, and in contrast to combinations of checkpoint inhibitors [117], adverse events were not compounded. This suggests that oncolytic viruses can be combined with checkpoint inhibition without a problematic decrease in safety.

### Oncolytic adenovirus trials

At the time of writing this review (March 2020), we did a search on clinicaltrials.org, resulting in 101 trial results. Limiting the search to “oncolytic adenovirus” phases I–II trials, we came up with 41 results; of these, 10 were completed and 15 recruiting. Sixteen different oncolytic viruses were used in these completed or recruiting trials. No active phase III clinical trials were found. Interestingly, 6 out of the 16 viruses have been posted recently, during 2019–2020, indicating growing interest and available funding for oncolytic adenovirus trials (Table 5).

**Table 5:** Sixteen oncolytic adenoviruses used in phase I–II trials that have been completed or recruiting (Mar-2020 clinicaltrials.org).

First posted	Last update	Oncolytic adenovirus (transgene)	Study title(s)	Indication(s)	NCT# identifier
2012	2019	ONCOS-102^a^ (GMCSF)	Completed: ONCOS-102 (Previously CGTG-102) for Therapy of Advanced Cancers Recruiting: (1) A Pilot Study of Sequential ONCOS-102, an Engineered Oncolytic Adenovirus Expressing GMCSF, and Pembrolizumab in Patients With Advanced or Unresectable Melanoma Progressing After Programmed Cell Death Protein 1 (PD1) Blockade (2) A Phase I/II, Safety Clinical Trial of DCVAC/PCa and ONCOS-102 in Men With Metastatic Castration-resistant Prostate Cancer	Solid tumors, melanoma, and prostate cancer	NCT03003676, NCT03514836
2012	2015	Delta-24-rgd	Completed: Safety Study of Replication-competent Adenovirus (Delta-24-rgd) in Patients With Recurrent Glioblastoma	Glioblastoma	NCT01582516
2013	2020	DNX-2401^b^	Completed: (1) Oncolytic Adenovirus, DNX-2401, for Naive Diffuse Intrinsic Pontine Gliomas (2) DNX-2401 With Interferon Gamma (IFN-γ) for Recurrent Glioblastoma or Gliosarcoma Brain Tumors (TARGET-I) (3) Virus DNX2401 and Temozolomide in Recurrent Glioblastoma (D24GBM) Recruiting: Oncolytic Adenovirus DNX-2401 in Treating Patients With Recurrent High-Grade Glioma	Gliomas	NCT03178032, NCT02197169, NCT01956734, NCT03896568
2013	2016	CELYVIR	Completed: Safety and Efficacy of Repeated Infusion of CELYVIR in Children and Adults With Metastatic and Refractory Tumors.	Solid tumors	NCT01844661
2013	2017	ICOVIR-5	Completed: Phase I Endovenous Administration of Oncolytic Adenovirus ICOVIR-5 in Patients With Advanced or Metastatic Melanoma	Melanoma	NCT01864759
2014	2019	VCN-01 (PH20 hyaluronidase)	Completed: A phase I Dose Escalation Study of Intratumoral VCN-01 Injections With Gemcitabine and Abraxane in Patients With Advanced Pancreatic Cancer Recruiting: Safety, Tolerability, and Efficacy of VCN-01 With Durvalumab in R/M Head and Neck Squamous Cell Carcinoma	Pancreatic cancer and head and neck	NCT03284268, NCT03799744
2014	2020	ColoAd1^c^	Completed: Mechanism of Action Trial of ColoAd1 (MOA) Recruiting: Chemoradiation With Enadenotucirev as a Radiosensitiser in Locally Advanced Rectal Cancer (CEDAR)	Colon, non-small cell lung, bladder, and renal cell	NCT02053220
2015	2019	CG0070 (GMCSF)	Completed: Safety and Efficacy of CG0070 Oncolytic Virus Regimen for High Grade NMIBC After BCG Failure (BOND2)	Superficial bladder cancer	NCT02365818
2020	2020	TILT-123^d^ (TNFalpha and IL-2)	Recruiting: TNFalpha and Interleukin 2 Coding Oncolytic Adenovirus TILT-123 During TIL Treatment of Advanced Melanoma	Melanoma	NCT04217473
2016	2020	LOAd703 (CD40L and 41BBL)	Recruiting: (1) Trial Investigating an Immunostimulatory Oncolytic Adenovirus for Cancer (2) LOAd703 Oncolytic Virus Therapy for Pancreatic Cancer (3) A Phase I/II Trial Investigating LOAd703 in Combination With Atezolizumab in Malignant Melanoma	Pancreatic, ovarian, biliary, colorectal, and melanoma	NCT03225989, NCT02705196, NCT04123470
2019	2020	ORCA-010	Recruiting: First in Man Clinical Study to Evaluate Safety and Tolerability of an Oncolytic Adenovirus in Prostate Cancer Patients.	Prostate cancer	NCT04097002
2017	2017	Ad5-yCD/mutTKSR39rep-hIL12 (IL-12)	Recruiting: Phase 1 Trial of Interleukin 12 Gene Therapy for Metastatic Pancreatic Cancer	Pancreatic cancer	NCT03281382
2017	2019	NSC-CRAd-Survivin-pk7	Recruiting: Neural Stem Cell Based Virotherapy of Newly Diagnosed Malignant Glioma (neural stem cells loaded with NSC-CRAd-Survivin-pk7)	Glioma	NCT03072134
2019	2020	NG-641 (CXCL9/CXCL10/IFNα)	Recruiting: First in Human Study With NG-641, an Oncolytic Transgene Expressing Adenoviral Vector FAP-TAc antibody together with an immune enhancer module (CXCL9/CXCL10/IFNα).	Epithelial tumors	NCT04053283
2016	2019	ADV/HSV-tk (HSV-tk)	Recruiting: SBRT and Oncolytic Virus Therapy Before Pembrolizumab for Metastatic TNBC and NSCLC (STOMP)	Breast and lung	NCT03004183
2019	2020	NG-350A (anti-CD40 ab)	Recruiting: First in Human Study of NG-350A (an Oncolytic Adenoviral Vector Which Expresses an Anti-CD40 Antibody) (FORTITUDE)	Epithelial tumors	NCT03852511

^a^ONCOS-102 was previously used in our ATAP treatment series as Ad5/3-D24-GMCSF and CGTG-102 [88]

^b^Previously known as Delta-24-rgd

^c^Also knowns as enadenotucirev

^d^TILT-123 is a double-armed virus designed for activation of T cells. It was designed based on human data from ATAP

### Use of oncolytic viruses in hematologic malignancies

Observations of hematological cancer regressions following virus infection were seen early last century [17, 18]. Of note, response evaluation was possible for hematological malignancies, by microscopy and cell counting, while this was not true for most solid tumors at the time. This might have caused overrepresentation of hematological cancer in early literature, due to observational bias. However, it cannot be denied that many reports suggested regression of hematological tumors after viral infections. As such, there is strong rationale to believe that oncolytic viruses could be used also in hematological malignancies. However, not all viruses are suitable for treatment of blood cell tumors. For example, adenovirus does not appear to be able to lyse white blood cells [118].

According to recent publications, growing interest towards oncolytic viruses is present also in the hematological field, as several viruses are being studied in preclinical settings [119]. However, only a few trials have been published [120]. A search from clinicaltrials.gov did not reveal any results for trials on “hematologic neoplasm” and “oncolytic viruses” (March 2020). However, a search for “multiple myeloma” and “oncolytic viruses” found two studies.

To conclude, to date, there have been few trials with oncolytic viruses in hematological cancers. Some noteworthy efforts include early phase trials with reovirus [121], measles [122], and vesicular stomatitis virus (VSV) in multiple myeloma [120]. The currently recruiting VSV trial (NCT03017820) also includes patients with relapsed acute myeloid leukemia and T cell lymphoma. No hematological trials with oncolytic adenovirus were found.

### Limitations and newer strategies to improve efficacy of oncolytic viruses

Arming with immunostimulatory cytokines has been one popular method to generate immunological synergy with the effects of oncolysis. Clinical benefit of this approach was seen in the phase III OPTiM trial where 1 in 6 patients achieved complete responses with the oncolytic virus talimogene laherparepvec. The median duration of these complete responses in the virus group was not reached and 8 out of 9 patients survived over 5 years [114].

Combining oncolytic viruses with T cell activating checkpoint inhibition can improve antitumor efficacy of oncolytic adenovirus therapy. Especially tumors with low amount of immunological cells—“cold tumors”—can be efficiently immune activated by oncolytic adenoviruses. This makes tumors “hot” and promotes the effects of checkpoint inhibitors [94, 123]. Clinical proof-of-concept for the efficacy of combining oncolytic viruses with checkpoint inhibitors has been presented [116, 124]. In a randomized phase II study (*n* = 198) a checkpoint inhibitor (ipilimumab) was combined with the oncolytic virus talimogene laherparepvec, showing objective responses in 39% of the patients, compared to 18% in the checkpoint inhibitor-only arm [116, 125].

In addition to melanoma, recent publications suggest clinical activity also in other cancers such as metastatic sarcoma where the same combination resulted in a 30% objective response rate in a single-arm trial [126]. Furthermore, the safety profile of these treatments has been good and oncolytic viruses do not seem to increase the rate of serious adverse events.

Although these combinations seem to yield increased potency and long-term benefits to some patients, not all benefit, and there is clearly a role for further improvement. Combining chemotherapy or radiation therapy in a rational way to improve treatment benefits, and even these conventional therapies seem to have an immunological component [27, 127]. However, clear clinical proof in support of this approach is currently limited.

Combining other therapies such as adoptive cell therapy or targeted therapies might also result in better treatments. However, we are still lacking much information about the immune effects in individual tumors. This insufficiency of knowledge makes it hard to understand which patients would benefit most of what kind of treatment combinations. This could be the greatest challenge in the field at the moment. Classic trial design is not well suited for understanding mechanisms on an individual tumor and patient level.

### TILT-123 studies

An ideal cancer treatment should be so good that most patients would clearly benefit while side effects should be tolerable. Our own contribution to this quest is a novel oncolytic adenovirus, designed specifically with T cells in mind. Ad5/3-E2F-D24-hTNFa-IRES-hIL2 (TILT-123) is based on the well understood and safe adenovirus serotype 5, but its fiber knob has been changed to a serotype 3 knob for enhanced penetrance to tumor cells [128]. The replication of the virus is strictly limited to cancer cells by dual control (E2F promoter and D24 deletion) and the potency of the virus is optimized by two transgenes, which were selected in a data-driven manner [95, 129]. Chimeric 5/3 adenoviruses also represent the best native T cell stimulator, among clinically relevant oncolytic adenoviruses [130].

The combination that emerged as the best approach for recruiting and activating T cells was interleukin-2 (IL-2) and tumor necrosis factor alpha (TNFa) [129]. IL-2 is required for T cell growth and survival and TNFa is a potent inducer of T cell trafficking and tumor apoptosis [129, 131–133]. In preclinical models, administration of cytokine-coding adenoviruses increased the antitumor efficacy of three forms of adoptive T cell therapy: T cell receptor (TCR)-engineered T cells [129], CAR T cell therapy [134], and tumor-infiltrating lymphocyte (TIL) therapy [128, 135].

This outcome resulted from the following: (1) improved infiltration of transferred cells induced by TNFa, (2) improved activity of cytotoxic T cells induced by IL-2, and (3) overall decrease of immune suppressive subsets including regulatory T cells (Tregs), MDSCs, and M2 macrophages in the tumor microenvironment [129]. Hamsters bearing pancreatic tumors treated with TILT-123 showed signs of improved antitumor efficacy as compared to animals receiving TIL therapy or TILT-123 alone [128]. Combination of IL-2 and TNFa coding adenoviruses and anti-PD-1 therapy also fostered long-term 100% survival in preclinical models bearing a melanoma tumor model [136].

Further studies with the virus demonstrated abscopal effect in non-injected tumors, showing the systemic nature of the immune response generated by local treatment [128]. Moreover, TILT-123 therapy in TIL-treated animals demonstrated protective immunity towards tumor re-challenge [128]. Importantly, this data demonstrates the potential of TILT-123 to fine-tune and overcome challenges of T cell-based adoptive cell therapy. The first trial with this advanced oncolytic adenovirus is now ongoing (NCT04217473).

## Conclusions

In summary, while preclinical data for oncolytic adenoviruses is impressive and clinical data have shown efficacy, more potent treatment strategies are needed to achieve long-term tumor control in patients. Thus, it is logical that several approaches are being utilized for improving the efficacy of oncolytic viruses. Hopefully, the efforts of the scientific and medical communities during multiple decades will lead to better treatment options in the near future. Better understanding of individual patient-tumor immune status will likely emerge and we will be able to treat advanced cancer patients better. It appears that oncolytic viruses will be part of future multimodality approaches. New treatments are urgently needed to help the 10 million cancer patients that are dying each year.

## Data Availability

Not applicable

## Abbreviations

5-FC: 5-fluorocytosine
ADCC: Antibody-dependent cell-mediated cytotoxicity
ATAP: Advancer Therapy Access Program
BCG: Bacillus Calmette–Guérin
CAR: Chimeric antigen receptor
CART: Chimeric antigen receptor T cells
CEA: Carcinoembryonic antigen
CXADR: Coxsackie- and adenovirus receptor
DAMP: Damage-associated molecular pattern
DCs: |Dendritic cells
DNA: Deoxyribonucleic acid
EGFR: Epidermal growth factor receptor
EMA: European Medicines Agency
FAP: Familial adenomatous polyposis
FDA: Food and Drug Administration
GMCSF: Granulocyte macrophage colony stimulating factor
hTERT: Human telomerase reverse transcriptase
IFN: Interferon
IL-2: Interleukin-2
MDSCs: Myeloid-derived suppressor cells
NK: Natural killer cells
PAMP: Pathogen-associated molecular pattern
PBMCs: Peripheral blood mononuclear cells
PSA: Prostate-specific antigen
RGD: Arginylglycylaspartic acid
TAMs: Tumor-associated macrophages
TCR: T cell receptor
TGFb: Transforming growth factor beta
TIL: Tumor-infiltrating lymphocyte
TNFa: Tumor necrosis factor alpha
Treg: Tegulatory T cell
